# Traffic Intersection Re-Identification Using Monocular Camera Sensors

**DOI:** 10.3390/s20226515

**Published:** 2020-11-14

**Authors:** Lu Xiong, Zhenwen Deng, Yuyao Huang, Weixin Du, Xiaolong Zhao, Chengyu Lu, Wei Tian

**Affiliations:** Institute of Intelligent Vehicles, School of Automotive Studies, Tongji University, Shanghai 201804, China; xiong_lu@tongji.edu.cn (L.X.); dengzhenwen@tongji.edu.cn (Z.D.); huangyuyao@tongji.edu.cn (Y.H.); 1733345@tongji.edu.cn (W.D.); 1651860@tongji.edu.cn (X.Z.); luchengyu@tongji.edu.cn (C.L.)

**Keywords:** monocular camera sensor, deep learning, intersection dataset, intersection re-identification, image matching

## Abstract

Perception of road structures especially the traffic intersections by visual sensors is an essential task for automated driving. However, compared with intersection detection or visual place recognition, intersection re-identification (intersection re-ID) strongly affects driving behavior decisions with given routes, yet has long been neglected by researchers. This paper strives to explore intersection re-ID by a monocular camera sensor. We propose a Hybrid Double-Level re-identification approach which exploits two branches of Deep Convolutional Neural Network to accomplish multi-task including classification of intersection and its fine attributes, and global localization in topological maps. Furthermore, we propose a mixed loss training for the network to learn the similarity of two intersection images. As no public datasets are available for the intersection re-ID task, based on the work of RobotCar, we propose a new dataset with carefully-labeled intersection attributes, which is called “RobotCar Intersection” and covers more than 30,000 images of eight intersections in different seasons and day time. Additionally, we provide another dataset, called “Campus Intersection” consisting of panoramic images of eight intersections in a university campus to verify our updating strategy of topology map. Experimental results demonstrate that our proposed approach can achieve promising results in re-ID of both coarse road intersections and its global pose, and is well suited for updating and completion of topological maps.

## 1. Introduction

Road intersection re-identification is an essential ability for human drivers. To arrive at the destination, a global driving route has to be planned. By re-identifying intersections on the planned route, drivers can decide the proper behaviors at different phases when traversing intersections. For instance, before entering the intersection, the vehicle should reduce the speed and select the correct lane according to the planned driving direction. Within the intersection, vehicles should take a correct motion and be alerted to environmental objects. When leaving the intersection, the vehicle recovers the instructed speed and continues to drive along the current road.

**Figure 1:**
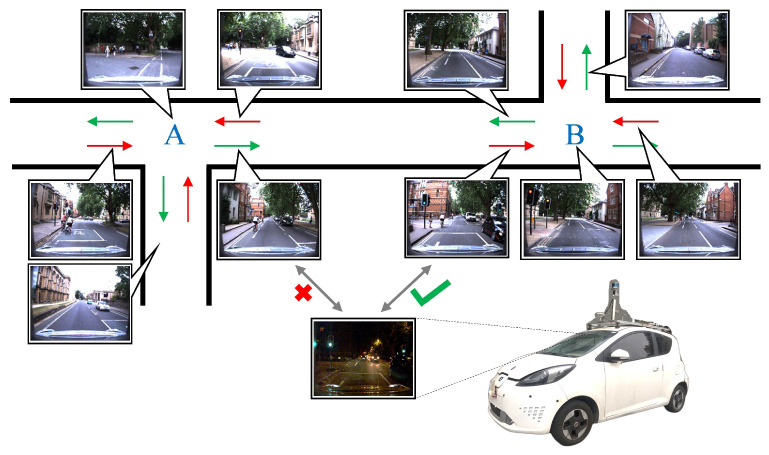
Traffic intersection re-identification for sparse localization of IV. Camera images captured at different positions in each intersection area are mapped and used to extract gallery features, which match the query image to achieve the sparse localization. The gallery images associated with the locations can make up a topological map. Image samples are from [6].

The same behavior decision at intersection also applies for automated driving. With the recurrence of deep learning, the road intersection re-ID task can be accomplished by leveraging image features (e.g., trees, infrastructure elements, road markings, traffic signs) and deep convolutional neural networks (DCNNs). By introducing the prior information of a digital map, the intersection re-ID can also provide a rough positioning of the vehicle when the intersection is detected and identified, as shown in Figure 1. Compared to simultaneous localization and mapping (SLAM), visual place re-identification [1] is unobligated to save a vast amount of point clouds or visual feature points, and thus is suitable for sparse localization. (In this work, we refer to the localization w.r.t. the topological map, which focuses on the pose relative to the intersection and the driving direction of vehicle.) In this paper, we argue that the required information in driving behavior decision for intelligent vehicles (IVs) such as fine intersection attributes and sparse positioning w.r.t. the intersection topological map can be achieved under a rational road intersection re-ID approach.

Due to the importance of road intersections, recent studies have been focused on representation of intersections by exploiting recurrent sequences [2], producing HD maps [3], analysis of driving behavior at intersections by tracking strategies [4], or planning horizons [5]. However, this research either barely considers the intersection into larger designated areas or insufficiently uses vehicular sensors to re-identify the intersection in conjunction with the topological map.

Monocular cameras are often considered as sensors for perception task solutions in the automotive industry. Compared with other vehicle-mounted sensors, such as LiDAR and radar, cameras can provide images with rich environmental semantic information and have a relative low production cost and high installation flexibility. Meanwhile, the training data and deep neural network structure (open source) from academic communities further improve the performance of image processing. Although machine learning based image processing has achieved significant progress in recent years, using a monocular camera sensor for intersection re-ID is fundamentally challenging for modern deep neural networks. As a sensor deficiency, the monocular camera has no depth information, resulting in the fact that the intersection geometry cannot be fully used. The complex traffic flow at the intersection can also interfere with the extraction of static environmental features. Currently, there is no framework designed for automated driving to complete multiple tasks including intersection classification, intersection recognition, and intersection re-ID with a monocular camera sensor. To the best of our knowledge, there are neither public datasets available that address the evaluation of road intersection re-identification in the traffic environment, which has further slowed down the related research.

To address the issues above, in this paper, we firstly introduce the intersection re-ID task formulation, which is ready to associate proper driving behavior decision under a given intersection topological structure. Secondly, we design the Hybrid Double-Level traffic intersection re-ID network as an end-to-end multi-task framework for the classification of intersection and its fine attributes, and the global localization in topological maps by monocular camera images. Thirdly, we present two intersection datasets: the RobotCar Intersection dataset, which is based on the prior work of [6] and covers more than 30,000 finely labeled intersection images in different time dimensions, and the Campus Intersection dataset, which contains 3340 panoramic images captured at eight intersections in the Tongji University campus. Finally, we validate the performance of our approach in the re-identification of road intersections and its global pose, and the performance of updating strategy for topology map on proposed datasets. The fully labeled datasets and corresponding PyTorch code of our framework will be online available (https://github.com/tjiiv-cprg/Road-Intersection-Re-ID).

## 2. Related Works

Image-based traffic intersection re-ID is the re-identification of intersections by establishing correspondence between intersection images captured by camera sensors. In a closed area, as shown in Figure 1, the intelligent vehicle firstly traverses all intersections, and saves intersection images with pose labels in the gallery. When arriving at the intersection again, the global location of vehicle and its route can be determined by query-gallery image matching. For example, in Autonomous Valet Parking [7], driverless cars will automatically drive to the corresponding intersection for pick-up service by image based intersection re-ID. For driving in an open area, the intersection re-ID approach is inherently able to recognize new intersections (e.g., by a missing match). Thus, the stored topological map can be further extended with a rational updating strategy.

Despite the importance, intersections are considered as the most complex part of the traffic road, mainly because vehicles from different driving directions will gather there and drive to their respective destinations, thus forming a complex traffic flow. As the hubs of transportation networks, intersections are very valuable for research. However, there are few studies on intersection re-identification based on vehicular onboard visual sensors, and related datasets are also scarce. Thus, in this section, we mainly summarize research from aspects of intersection detection, visual place recognition, and visual place re-ID datasets, which are most relevant to our topic.

### 2.1. Intersection Detection

Detection of intersections has been an interest point for scholars since the last decade. Kushner et al. [8] firstly presented road intersection detection methods respectively based on monocular camera and range scanner. They used road boundaries and height profiles to determine the best match by a minimum goodness-of-fit measure. As the superiority of deep neural networks emerges, Bhatt et al. [2] proposed an end-to-end Long-term Recursive Convolutional Network (LRCN) and considered intersection detection as a binary classification task on the frame sequence. In the work of Bhattacharyya et al. [9], a method of spatial-temporal analysis of traffic conditions at urban intersections based on stereo vision and 3D digital maps was introduced. The depth cues of each pixel effectively provide more accurate intersection detection. Habermann et al. [10] proposed to detect road intersections based on 3D point clouds. Three classifiers, including support vector machine (SVM), AdaBoost, and artificial neural network (ANN), are used for classification of intersections.

In non-visual sensor based intersection detection, Xie et al. [11] detected intersections indirectly from common sub-tracks shared by different global navigation satellite system (GNSS) traces. Local distance matrices, image processing techniques, and Kernel Density Estimations are used to identify the intersection. Based on remote radar images, Cheng et al. [12] detected different types of road intersection in two stages: global area detection and local shape recognition. However, intersection detection by non-visual sensor typically takes a long observation time.

### 2.2. Visual Place Recognition

A cognitive map [13] interprets a distinctive place according to the sensory information such as place signature and place description. To distinguish between images, hand-crafted local feature descriptors such as scale-invariant feature transform (SIFT) [14], speed-up robust features (SURF) [15], and features from accelerated segment test (FAST) [16] have been used in image matching. With hundreds of local features extracted from images, the bag-of-words model [17] collects all local features into a vocabulary and the frequency of each word can be used as distinctiveness criterion for similarity evaluation between two images. Global place descriptors based on color histograms [18] and Principal Component Analysis (PCA) [19] are also used in early localization systems. Presently, the Gist [20], as a 512-dimensional feature, is extracted from an image using Gabor filters at different orientations and different frequencies. This approach is widely used in global place recognition [21].

In addition to hand-crafted approaches, the neural network is also introduced to solve signature verification as an image classification or matching problem. Chen et al. [22] trained a simple and lightweight ConvNet to classify each place image into place labels. The Siamese network [23], consisting of twin networks to search matched image pair, is optimized for place recognition in [24]. As the weights of the twin networks are shared, output features of two distinct images are computed in the same metric which is essential to similarity calculation. Such a structure is widely used in re-identification of persons [25] and vehicles [26]. However, differing from common approaches for pedestrian or vehicle re-ID, the task for intersection re-ID focuses on the complex road intersections, which involve information from a wide traffic scene. The intersection re-identification network needs to be able to automatically extract static background features, instead of focusing on the foreground targets, e.g., pedestrians and vehicles. These existing methods for pedestrian or vehicle re-ID cannot be directly applied to intersection re-identification.

### 2.3. Visual Place Re-ID Datasets

One of the related datasets is the Alderley Day/Night Dataset [27], which was captured along a fixed route in two different conditions: sunny daytime and rainy nighttime. The Ford Campus Vision and Lidar Dataset [28] recorded both time-registered laser points and images, with the vehicle driving along several large and small-scale loops. The images in the FABMAP Dataset [29] were collected from cameras mounted on both sides of a robot pan-tilt. The image collection was triggered every 1.5 m (on the basis of odometry). The St Lucia Multiple Times of Day [30] collected visual data with a forward facing webcam attached to the roof of a car. In total, ten subdatasets were captured at five different periods of daytime in a two-week interval. The CMU Dataset [31] recorded images in sequences during one year from the urban, suburban, and park by two front-facing cameras, pointing to the left/right side at approximately 45 degrees. However, the mentioned datasets are collected along trajectories which did not contain traffic intersections, or their intersection images lack for environmental diversity.

With an improved diversity, the Oxford RobotCar Dataset [6] contains 20 million images collected from six vehicular cameras along a fixed route (including more than 25 obvious intersections) during a period of more than one year (per two weeks on average). All weather conditions, including sunny, overcast, rainy, or snowy weather in day and night, enable researchers to investigate long-term place re-identification in real-world and dynamic urban environments. However, this dataset can not be directly used in intersection re-ID due to the lack of detailed labels of traffic intersection.

Although there are many research and datasets related to intersection detection and visual place recognition, due to the essential difference, there is little academic work to address the re-identification of intersection and its attributes. In order to bridge the gap, we present the Hybrid Double-Level network and two corresponding datasets for this task.

## 3. Traffic Intersection Re-Identification Approach

### 3.1. Task Formulation

**Figure 2:**
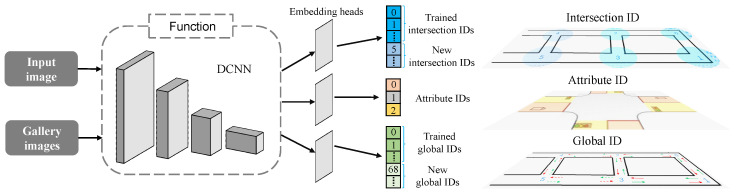
Multi-task of traffic intersection re-identification, which consists of determining the intersection ID, attribute ID, and global ID. The attribute ID denotes the interaction between ego-car and intersection, with 0 indicating at the entrance of the intersection, 1 for inside the intersection, and 2 for at the intersection exit. The intersection IDs and global IDs respectively represent different intersection areas and global poses (assigned with information of location and driving direction). Both of them can be partitioned into existing gallery IDs and new IDs.

The visual sensor based intersection re-ID task in this paper is defined as the multi-task including classification of intersection and its fine attributes, and the determination of global vehicle pose. While the intersection identity can be used in coarse localization, its attribute identity denotes the interaction between the ego-car and the intersection, and the global identity determines the global pose in the map (shown in Figure 2). Furthermore, new intersections should also be recognized when driving in an open area. These are the main characteristics different from conventional tasks of intersection detection and visual place recognition.

Mathematically, we define the set of gallery images from a closed area as $X={\left\{x_{i}\right\}}_{i=1}^{N}$. For each gallery image (actually the image feature) $x_{i}$ in this set, we map it to an ID tuple by a function *f* interpreted as

(1) $$f:x_{i}\to \left(u_{i},v_{i},w_{i}\right)\in U\times V\times W,$$

where $\left(u_{i},v_{i},w_{i}\right)$ represents the tuple of intersection ID, attribute ID and global ID of $x_{i}$. In addition, U×V×W represents the space of all valid intersection ID tuples. For a query image $\tilde{x}$, it can also be mapped to an ID tuple $\left(\tilde{u},\tilde{v},\tilde{w}\right)$ by function *f*. Thus, the intersection re-ID task in a closed area can be formulated as searching the optimal mapping function *f* by minimizing the classification error $\epsilon (\left(\tilde{u},\tilde{v},\tilde{w}\right),\left(u_{i},v_{i},w_{i}\right))$ with the assumption of groundtruth ID tuple as $\left(u_{i},v_{i},w_{i}\right)$. For an open area, if the query image $\tilde{x}$ represents a new intersection, the function *f* should be able to recognize it and assign the image with a new ID tuple $\left(u_{⊥},v_{i},w_{⊥}\right)$, interpreted as:

(2) $$f\left(\tilde{x}\right)=\left\{\begin{array}{cc} (u_{i},v_{i},w_{i}), & if\;\tilde{x}=x_{i}\\ (u_{⊥},v_{i},w_{⊥}), & otherwise \end{array}.\right.$$

Note that the attribute space V is unchanged in the task. For updating the topological map, the new intersection image along with its ID tuple should be added to the database, respectively yielding the extended gallery set $X_{ex}=\tilde{x}\cup X$ and extended ID tuple space $U_{ex}\times V\times W_{ex}=\left(u_{⊥},v_{i},w_{⊥}\right)\cup \left(U\times V\times W\right)$.

In this work, we interpret the function *f* with a DCNN architecture, which is introduced in Section 3.2. The mixed loss training of our network is explained in Section 3.3. In addition, the updating strategy of topology map is proposed in Section 3.4.

### 3.2. HDL Network Architecture

We propose a Hybrid Double-Level (HDL) network for traffic intersection re-identification to relieve the heavy burden from saving a vast amount of data in normal image matching. The proposed framework aims to learn a DCNN that can determine coarse classes like the intersection ID and attribute ID. The global ID is taken as a fine class, which is predicted by the similarity score between the query image feature and gallery image features.

**Figure 3:**
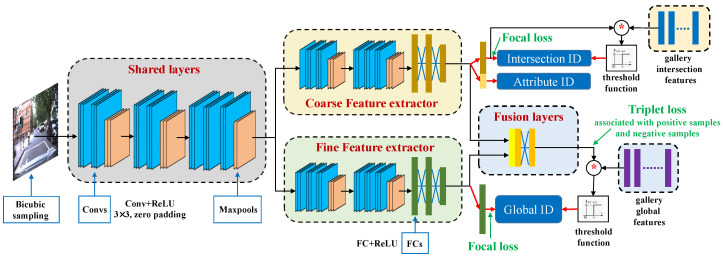
The proposed hybrid double-level network consists of two branches. The coarse features are concatenated into the mainstream to provide different level information for the generation of final feature. The similarity between the query feature and gallery features determines the global pose by threshold function. A coupled cluster losses help to train the network with three steps of forward propagation.

For the HDL network structure, inspired by the work [26], we exploit two DCNN branches to accomplish different tasks in traffic intersection re-identification, as depicted in Figure 3. In the proposed network, we first map the input image of three color channels to a canonical dimension using bicubic sampling and normalization. This normalizes the image scale while maintaining its aspect-ratio. The pre-processed image is represented as $I\in R^{H\times W\times 3}$ with the height *H* and width *W*. The proposed network is composed of one block of shared layers and two subsequent branches. The shared layers totally consist of seven convolutional filters of size 3×3 with ReLU activation layer, and three MaxPooling layers. The input image is firstly fed into shared layers to generate features for following two branches. The two DCNN branches are mainly for feature extraction in different scales: coarse and fine features. Each feature extractor contains convolutional blocks and fully connected blocks which are integrated with ReLU and dropout operation. We impose supervision on the coarse features to produce two embedding heads [32] for the classification of intersection and its attributes. The fine features with the same size are derived from the fine feature extractor and concatenated with coarse features as the input of a block of fusion layers. Note that the weights of both feature extractors are not the same. The fusion layers aim to fuse image information of different scales to represent global features of the intersection using a fully connected network. We perform the final embedding head to determine the global pose. The generation of coarse feature and global feature are respectively interpreted as

(3) $$F_{c}\left(I\right)=\sigma_{c}(Conv_{c}\left(\sigma_{s}\left(Conv_{s}\left(I\right)\right)\right),$$

(4) $$F_{f}\left(I\right)=\sigma_{f}(Conv_{f}\left(\sigma_{s}\left(Conv_{s}\left(I\right)\right)\right),$$

(5) $$F_{g}\left(I\right)=\sigma_{g}\left(Conv_{g}\left(F_{c}\left(I\right)⊕F_{f}\left(I\right)\right)\right),$$

where $F_{\left\{c,f,g\right\}}$ denotes generated feature from corresponding branches. The subscript *c* indicates coarse feature, *f* is for fine feature, and *g* denotes global feature. $Conv_{\left\{c,f,g\right\}}$ represents the corresponding convolutional layers and $\sigma_{\left\{c,f,g\right\}}$ represents corresponding activation function at each stage. ⊕ is the element-wise concatenation operation.

### 3.3. Mixed Loss Training

Since multiple tasks should be finished in our network, we strive to extract similar static background features from images of the same class as well as to distinguish the features between subclass images within one super-class. Such a task to recognize the subtle differences between highly similar images is called as the fine-grained image recognition. Moreover, to address the imbalance of training samples, we fuse a couple of loss functions to accelerate the training convergence and realize the multi-task.

In our network, we firstly introduce the Focal loss [33] based on the cross-entropy loss function of binary classification, which makes the network optimization less influenced by the imbalance of training samples. Both loss functions are interpreted as below:

(6) $$\ell_{cross-entropy}=\left\{\begin{array}{cc} -\log\tilde{y}, & if\;\;\;\;y=1\\ -\log(1-\tilde{y}), & if\;\;\;\;y=0 \end{array}\right.,$$

(7) $$\ell_{focal}=\left\{\begin{array}{cc} -\alpha {\left(1-\tilde{y}\right)}^{\gamma }\log\tilde{y}, & if\;\;\;\;y=1\\ -\left(1-\alpha \right){\tilde{y}}^{\gamma }\log\left(1-\tilde{y}\right), & if\;\;\;\;y=0 \end{array}\right.,$$

where $\tilde{y}$ is the output of activation function. *y* is ground truth label for binary classification. α and γ are hyperparameters for adjusting the imbalance of training samples. Compared with the standard cross entropy loss, the focal loss introduces an influence factor γ>0 to reduce the loss proportion of easy samples, as shown in Equations (6) and (7), so that the network pays more attention to samples which are difficult to be classified. Here, we use the focal loss in each branch to evaluate the identity between the instances represented by both images.

Additionally, we employ the triplet loss [32] in our network training. Different from the classification losses, the triplet loss enables the network to project raw images into an embedding space in which images of different categories can be separated by a certain margin. By this loss, the dimension of network output layer can be smaller than the number of categories which results in output dimension reduction. The triplet loss can also be applied to the problem of fine-grained identification, especially in intersection re-identification. To better train our proposed network, we use the triplet loss to evaluate the subtle difference among intersection images of different global IDs. Moreover, we use an online training strategy to acquire negative samples from the training batch. Different from the traditional L1 or L2 distance, we use the cosine distance to measure the embedding distance, as shown below:

(8) $$\ell_{triplet}=\sum_{n}max\left\{f_{sim}\left(I^{q},I^{n}\right)+M-f_{sim}\left(I^{q},I^{p}\right),\;0\right\},$$

(9) $$f_{sim}\left(I^{q},I^{n}\right)=\frac{F_{g}\left(I^{q}\right)*F_{g}\left(I^{n}\right)}{∥F_{g}\left(I^{q}\right)∥∥F_{g}\left(I^{n}\right)∥}\;\;,$$

where $f_{sim}$ is similarity function. $I^{q}$ is the input tensor of query image. $I^{p}$ is a positive sample with the same global ID. $I^{n}$ is a negative sample with a different global ID. * is the inner product. *M* represents the expected margin between inter-classes images and *n* represents the image number of a training batch. $∥\cdot ∥$ denotes the L2 norm of feature vector.

### 3.4. Updating Strategy of Topology Map

**Figure 4:**
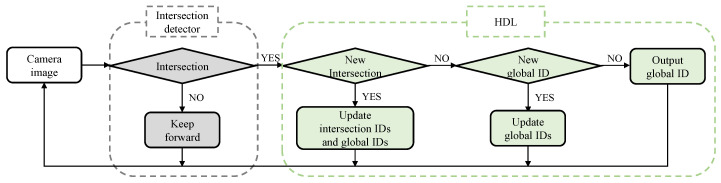
Updating strategy of intersection topological map. The system first perceives if the vehicle encounters an intersection. For a positive detection, the system checks if the image represents a new intersection or not. For a new intersection, the image will be assigned with new intersection and global ID. Both the image and its ID labels will be added into the database.

When driving in an open area, vehicles will often run into new intersections. How to add information of new intersections into the preset topological map is essential for autonomous vehicles driving in a real traffic environment. In this regard, we propose an updating strategy for the intersection topology map, as shown in Figure 4. This strategy is decomposed into two steps. In the first step, camera images are processed by conventional intersection detection approaches (as introduced in Section 2) to determine whether it represents an intersection or not. If it is a straight road, the vehicle continues to drive along the planned path. When an intersection is detected, the system determines whether it is currently at a new intersection or at an existed intersection by comparing the similarity of query image feature with gallery image features.

For an image of new intersection, a new intersection ID and a new global ID will be assigned to this image. For an existed intersection, only the global ID will be updated if the current lane or direction is not matched with the gallery. Furthermore, the new image along with its labels will be added into the database. Note that, since the interaction between intersection and vehicle does not depend on the intersection recognition result, only the label space of intersection ID and global ID can be changed.

## 4. Proposed Intersection Datasets

### 4.1. RobotCar Intersection Dataset

**Figure 5:**
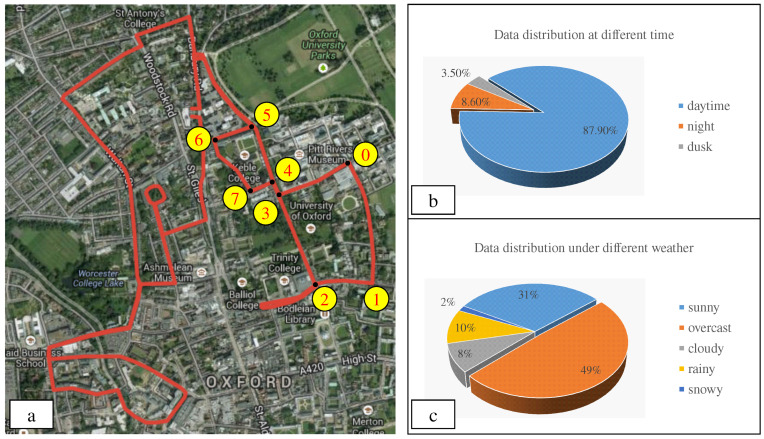
Dataset information. (**a**) the selected intersections (marked with yellow number) on the map [6]. (**b**) data distribution at different time. (**c**) Data distribution under different weather.

Our proposed RobotCar Intersection dataset is based on the prior work RobotCar [6], which is captured in the central area of Oxford by driving vehicle several times along a fixed route. In our dataset, to simulate the habits of human drivers, we choose images of the front-view camera (Bumblebee XB3, CCD, only $66^{\circ }$ Horizontal Field of View,) for intersection re-identification. Images of eight road intersections in a closed region (over 30,000 images with a resolution of 1280×960) are selected to build our dataset, as shown in Figure 5a. Inheriting the characteristics of the original RobotCar dataset, the selected images are with environment conditions of different seasons (from May 2014 to November 2015), time (day/night/dusk), weather (sunny/overcast/cloudy/rainy/snowy), etc. Detailed statistics about these images are shown in Figure 5b,c.

**Figure 6:**
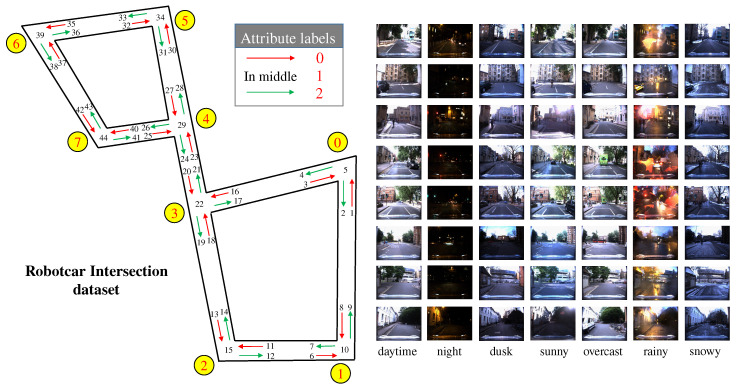
Examples of intersection labels. We label each image with an intersection ID, an attribute ID and a global ID. The ID arrangement is determined by the designated driving route in clockwise order. Image samples in different weathers are from [6].

To add refined attribute labels of intersection to the new dataset, firstly, we determine different traversing phases w.r.t. the relative location between the vehicle and the intersection. The entering phase is determined when the vehicle is located in front of the diversion/stop line at the entrance road of the intersection; the crossing phase is defined when the vehicle is located within the intersection, i.e., between the stop lines of the entrance and the exit road; The leaving phase is determined when the vehicle enters the exit road and completely leaves the intersection. These phases and their associated moving directions are considered as intersection attributes, which are along with the intersection ID assigned to corresponding images, as shown in Figure 6.

Furthermore, we assign each image with an additional global traversing pose ID for better recording the traversed route of the vehicle. Thus, each intersection image is labeled with three kinds of IDs. As shown in Figure 6, the intersection ID is only to identify the intersection location (0–7). The attribute ID of the intersection indicates the interactive relationship between the vehicle and the intersection: the label for entering the intersection is 0, the intersection crossing is labeled as 1, and the intersection exiting is denoted as 2. The global ID represents that the vehicle is at the designated location with designated driving direction. The extracted images are stored according to recording dates, and marked with the weather information. Corresponding labels are also stored in a similar way.

From the examples in Figure 6, it can be seen that the image texture information is the most abundant in cloudy days. The images in sunny weather are easily affected by exposure and halo. The earth ground in images are obviously different in rain and snow weather. The texture information of night images is less than that in the day. All of these facts make the intersection re-ID task on this new dataset challenging.

### 4.2. Campus Intersection Dataset

**Figure 7:**
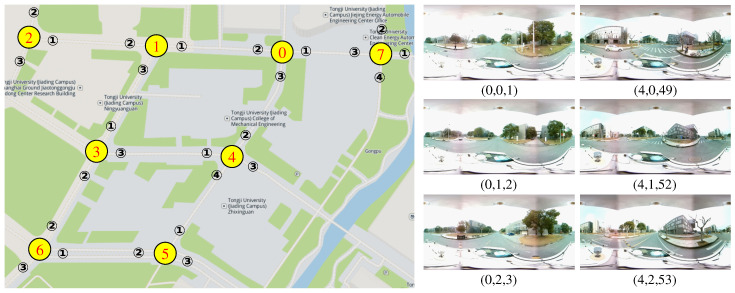
Selected campus intersections and labeled examples. We label intersection IDs according to the driving route and the global IDs in designed order (e.g., clockwise). Each image is labeled with a tuple of (intersection ID, attribute ID, global ID).

The panoramic image has a wide perception area in the surrounding environment, which can also be used for traffic intersection re-identification. In order to explore the performance of our proposed framework on different camera models, we provide the “Campus Intersection” dataset which consists of 3340 spherical panoramic images from eight intersections in the Jiading campus of Tongji University, as shown in Figure 7. The images are captured by a panoramic camera setup introduced in work [34] with a resolution of 1800×900. The panoramic camera is formed by two fisheye cameras (S-YUE-U801, CMOS, $210^{\circ }$ Horizontal Field of View), which are installed back-to-back in this case.

We label the Campus Intersection images with the same method as previously introduced. Intersection IDs are determined according to the driving route, and the global IDs are labeled with designed order (e.g., in clockwise order). Note that the intersection ID and attribute ID cannot determine the global position of the vehicle on the topological map, while only the global ID corresponds to the global pose of the vehicle.

## 5. Experiments

In this section, we evaluate the performance of proposed HDL network architecture on both the RobotCar Intersection and the Campus Intersection datasets, with the motivation of exploring its robustness against different camera models. The experimental results are evaluated by using the prevailing metric of accuracy, margin, precision and recall value, as shown below:

(10) $$Accuracy=\frac{\sum_{N}\left(t_{i}\right)}{N},\;\;\;t_{i}=\left\{\begin{array}{cc} 1, & if\;f_{sim}\left(I_{i}^{q},I_{i}^{p}\right)>M+f_{sim}\left(I_{i}^{q},I_{i}^{n}\right)\\ 0, & otherwise \end{array}\right.,$$

(11) $$Margin=\frac{\sum_{N}\left(f_{sim}\left(I_{i}^{q},I_{i}^{p}\right)-f_{sim}\left(I_{i}^{q},I_{i}^{n}\right)\right)}{N}\;\;,$$

(12) Precision=TP/(TP+FP),

(13) Recall=TP/(TP+FN),

where TP, FP, and FN respectively denote the number of true positives, false positives, and false negatives. *N* represents the number of query images. If the similarity between query image and gallery images is larger than a pre-defined threshold, the global ID of the most similar gallery image is assigned to the query image.

### 5.1. Training Configuration

For training, the weights of our proposed network are initialized by the pre-trained layers of a VGG16 [35] network. In detail, we use the first seven convolutional layers of the pre-trained VGG16 network to initialize the shared layers. The initial weights of each feature extractor are from the remaining layers of the pre-trained network. Weights of the fusion layer are initialized by a uniform distribution $U(-\sqrt{1/k},\sqrt{1/k})$, where *k* is the input tensor size. Additionally, our proposed model is trained with respect to a combination of detection loss and embedding loss, as follows:

(14) $$L_{Total}=\lambda_{c}\ell_{c}^{F}+\lambda_{f}\ell_{f}^{F}+\lambda_{g}^{F}\ell_{g}^{F}+\lambda_{g}^{T}\ell_{g}^{T},$$

where $\ell_{c}^{F}$, $\ell_{f}^{F}$ and $\ell_{g}^{F}$ respectively denote the Focal loss of coarse, fine, and global features. $\ell_{g}^{T}$ denotes the triplet loss of global features. The weights $\lambda_{c}$, $\lambda_{f}$, $\lambda_{g}^{F}$ and $\lambda_{g}^{T}$ for each loss are empirically set to 0.5, 0.5, 0.5, 1, respectively.

In the training process we use an online triplet mining method to select the most dissimilar positive sample and the most similar negative sample in the embedding space from the training batch. All networks are trained using the stochastic gradient descent (SGD) optimizer with an initial learning rate of 0.001, a weight decay of $1\times 10^{-8}$ and a momentum of 0.9. The Focal loss is adopted with parameters α=0.25 and γ=2 (under such parameters the focal loss can work best [33]). The batch size is set to 4, which means we need to feed 4×5=20 images in each training iteration (which is a compromise between network convergence speed and GPU computing load). All networks in our experiments are implemented by PyTorch. The evaluation environment is with an Nvidia GTX 1080Ti GPU, an Intel Xeon E5-2667 CPU of 3.2 GHz and a memory of 16 GB.

### 5.2. Data Augmentation

**Figure 8:**
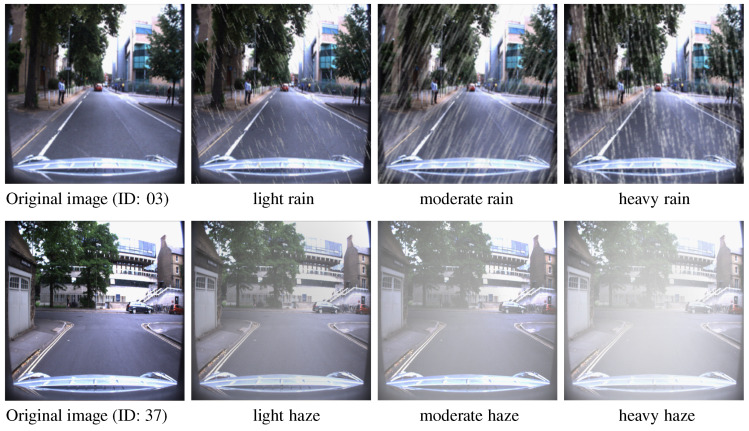
Intersection image with data augmentation by a nonlinear raining model (**first row**) and haze model (**second row**). Images samples are from [6].

To enhance the generalization ability of trained networks especially on the illumination change, vehicle sway, camera extrinsic and intrinsic parameter change, we deploy data augmentation methods including random illumination, affine transform, and perspective transform. Since vehicles may also drive in extreme meteorological environments such as the rain or haze, data augmentation by adding rain and fog in images will improve the network performance under the extreme weather. Here, we introduce a nonlinear raindrop model [36], as shown below

(15) $$I_{r}=\beta_{r}I_{M}+\left(1-I_{M}\right)⊙I_{c},$$

where $I_{r}$ is a rainy image. $I_{c}$ is the clean background image. $I_{M}$ is the rain map and $\beta_{r}$ is the brightness of raindrops. ⊙ represents channel-wise operation between $I_{M}$ and $I_{c}$. In order to simulate natural raindrops, we randomly set the raindrop falling direction within the range of $\pm 30^{\circ }$, and the transparency of raindrops from 0.7 to 1.0, as shown in the first row of Figure 8.

For haze simulation, without the depth information, it is tricky to generate haze with only a single image. Here, we follow the classical haze generation [37] of atmospheric scattering model as

(16) $$I\left(x\right)=J\left(x\right)t\left(x\right)+\beta_{h}A\left(1-t\left(x\right)\right),$$

where I(x) is the observed hazy image value at pixel *x*. J(x) is the haze-free scene radiance to be recovered. $\beta_{h}$ is the brightness of the haze. *A* denotes the white atmospheric light. t(x) is the transmission matrix which is set with nonlinear proportion of pixel distance between each pixel and vanishing point. The density and brightness of haze is adjusted smoothly from 0.7 to 1.0, as shown in in the second row of Figure 8.

### 5.3. Evaluation on RobotCar Intersection

We divide the RobotCar Intersection dataset into two subsets according to the recording date. The first 60% of the total dataset, containing 21,968 images, are used for training. The remaining 40%, containing 14,620 images, are used for testing. The training data covers exactly a full year, allowing the network to fully learn the characteristics of data in different seasons and weather. The testing data are from a time period completely different from the training data.

In this experiment, we evaluate different network structures and augmentation methods. As mentioned in Section 3.2, we keep the first three blocks as the shared layers and the last three blocks as a feature extractor to build two branch networks (2B). Additionally, we integrate the triplet loss (TRI) instead of the single focal loss (FOC) (only for estimating global ID) to reinterpret the image classification problem as an image re-identification problem. Furthermore, image generation by rain and haze model which forms data augmentation (AUG), are also verified in our experiments. Totally, networks with five different configurations (see Table 1) are trained. Moreover, we also compare the results of the one branch networks (1B) which cancels the coarse feature extractor from the proposed HDL.

**Figure 9:**
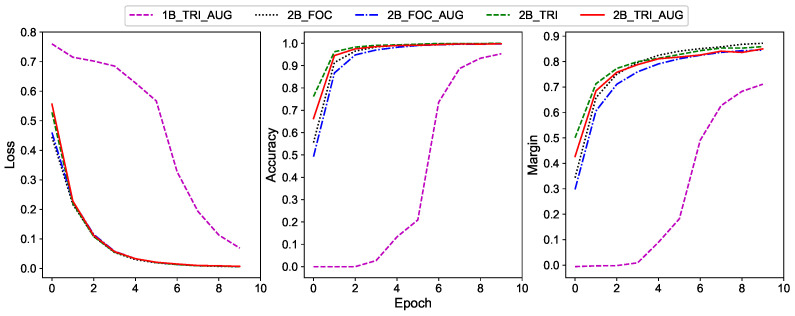
Loss, accuracy, and margin w.r.t. epoch number in training for different network structures and augmentation methods.

Curves of loss, accuracy, and margin w.r.t. the epoch number are shown in Figure 9. It is obvious that the utilization of two branch networks can make the loss converge faster than those with only one branch, and the accuracy and margin curves behave similarly. This means that the coarse features indeed help the network to learn the fine features. It can also be seen that employing the triplet loss can accelerate the network learning, which demonstrates the effectiveness of triplet loss. In contrast, training with the data augmentation has larger loss value but less accuracy and margin before the network converges. This can be attributed to the fact that both data augmentation approaches increase the diversity of training data and make the learning process more complicated.

**Figure 10:**
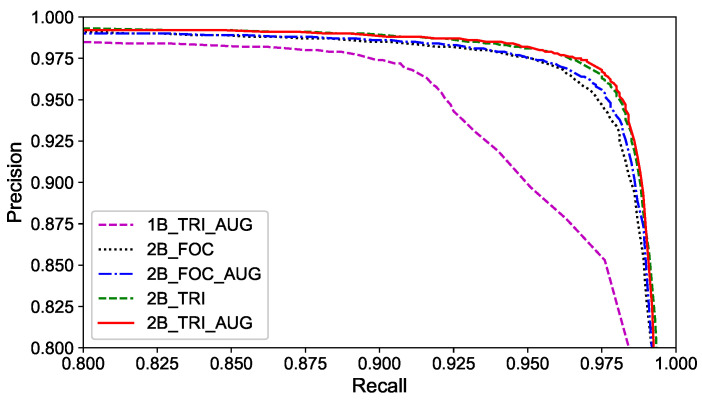
The PR curve.

In experiments, the precision–recall (PR) curves of compared methods are depicted in Figure 10. From the curves, following points can be observed: (1) The method proposed in the paper can achieve high precision on intersection re-identification. (2) The performance of hybrid double-level network is better than that of the single-branch network, which is mainly because the HDL network can integrate coarse-grained features into the mainstream and provide semantic information of different scales. (3) The data augmentation also brings a minor performance gain, which demonstrates the positive effect by improving the diversity of training data.

The quantitative experiments are designed with three additional baseline methods, i.e., the bag-of-words (BOW) [17], which is frequently used in visual SLAM to determine if a vehicle revisits the starting position in a loop closure detection, ConvNet [22] and SiameseNet [24], which are widely used in image classification or image matching for visual place recognition. For a better impression of network performance, we report the precision of evaluated methods w.r.t. different environmental conditions in Table 2. Normally, because of the low-light, image features of intersections strongly degrade at night compared with the daytime. In addition, the road texture in rainy and snowy weather has lower contrast, and the light scattering of raindrops also forms light spots on the image. All of these phenomena mentioned have severely affected the performance of the traditional bag-of-words method. However, the precision of deep learning based methods fluctuates much less. We owe it to the fact that the neural network can automatically learn more distinctive and robust intersection information from the image feature embedding space than the hand-crafted features used in traditional methods.

From the results in Table 2, our proposed methods surpass the three baseline methods in the test on RobotCar Intersection data. This depends on the reasonable network structure and mixed loss. It can also be seen that the training with data augmentation fused with rain and haze model can further improve the precision of intersection re-identification. This is because the training and test dataset contains a small number of images captured during light rain or on a wet road. Training with augmented image data from rain and haze model alleviates the imbalance of training samples. This also shows that the utilized rain and haze model can well simulate the image noise by rain and haze in the real traffic environment. For a further analysis, we also add generated rain and haze images (which are different from the images used in training) to the test data and re-evaluate each network. The dataset becomes more difficult, which can be seen from the decreased precision results shown in Table 3. However, the networks trained on augmented data still obtain better re-identification results on the augmented test data.

Since dynamic objects can also interfere with the captured intersection scene by camera sensor, we conduct further experiments to evaluate the performance of proposed approach in different traffic flows. First, we assign intersection images of test set with labels which indicate different traffic flow: 0 for less surrounding dynamic objects, and 1 for more dynamic objects. The precision of compared methods are shown in Table 4 and Table 5.

Comparing results in Table 4 and Table 5, it is obvious that the accuracy of all intersection re-ID methods is reduced in high traffic flow with more dynamic traffic participants. This is mainly because the surrounding dynamic targets occlude part of the image of static environmental background, which makes some landmark signs, e.g., road markings, traffic facilities, surrounding buildings, not well recognized, and thus causes the mis-identification of intersection images. However, as seen from the comparison results, our proposed approach only exhibits a small accuracy decline and still outperforms other compared approaches.

In addition, the test results of BOW [17] show that the images captured on overcast days can be better recognized in different traffic flow. This indicates that the intersection images in overcast condition have richer textures than in other conditions, since the training of BOW [17] is to extract all point features in images. Moreover, the network by the training with rain-haze augmentation (2B + TRI + AUG), in some extreme environmental conditions, such as in the rain, can obtain better re-identification results. This is mainly because the rain-haze augmentation imposes noise disturbance (which resembles the real scene) on the network. This enables the network to extract more robust features from the static environmental background.

### 5.4. Results on Campus Intersection

In this chapter, we use the Campus Intersection dataset to accomplish two main tasks: (1) verifying the effectiveness of the proposed method under panoramic camera modality; and (2) verifying if the double-level network could be used to detect new intersection images and expand the topological map. Hence, we divide the Campus Intersection dataset into two subsets according to the intersection ID. The 60% data of the first five intersections, containing 1456 images, are used to train the network. The remaining 40% images of the first five intersections, containing 995 images, are used to test the network on panoramic intersection images for existed intersection re-ID. The images of the last three intersections, containing 897 images, build the other subset of new intersection images and are used for the re-ID task of new intersections.

Due to minor changes in weather condition, in this experiment, we solely employ the double-level network with triplet loss and without rain-haze augmentation (2B + TRI). Similarly, images of each global ID are randomly selected from training images to form gallery images. Each testing image then is compared with each gallery image by the similarity function, and we calculate the accuracy of re-identification. The high precision results from the first row of the Table 6 show that the proposed network can well detect existed intersections with their intersection ID and attribute ID in the panorama image modality; meanwhile, it can re-identify the intersection with the global ID.

Moreover, according to the expansion strategy of intersection topological map, the proposed network structure must be able to detect new intersection images, which can be categorized into two types: the first type of image is from an existed intersection but at a new lane or in a new direction. Thus, it is with a high intersection ID similarity but with a low global ID similarity. The second image type is from an entire new intersection, and thus with low similarities for both kinds of IDs. In this part of experiment, we use the global intersection ID to detect new intersection images. To determine the optimal threshold of similarity function, we first obtain the histograms by summarizing the similarity values between all query images (existed IDs and new IDs) and gallery images. Then, we set the trough of the histograms as the threshold, which is 0.87 for best detect precision of global ID. By adopting the optimal parameter settings in our experiment, the detection precision of attribute ID is 0.855 and for global ID, it is 0.990, as shown in the second row of Table 6.

For images with the same new global ID, we randomly select one of them and add it to the gallery. Then, we run the re-ID test with the new gallery again. The results are shown in the third row of Table 6 and demonstrate that our proposed intersection re-ID network can successfully handle images of new IDs. Since the attribute ID is from the classification of coarse features, it will be consistent with the result of the second row. Now, the gallery is updated by the images of new IDs, then forming larger closed-paths. All the query images in the larger area will be tested and shown in the forth row of Table 6. All the results in Table 6 show that our proposed network are suitable for road intersection re-identification.

In the intelligent driving system, real-time performance is usually an important performance parameter of the designed network model. In the experiments, our approach can achieve a fast image processing speed of 16 frames per second (FPS). It can be applied in real-time autonomous driving tasks.

## 6. Conclusions and Future Work

In this paper, we propose a Hybrid Double-level network to solve an important but not well explored problem: traffic road intersection re-identification. We exploit a two-branch DCNN to accomplish multi-task including classification of intersection and its fine attributes, and global localization in topological maps. Compared with the traditional methods, our deep learning-based method avoids storing vast amount of feature points and has higher accuracy of re-identification. Experimental results demonstrate that our proposed network can complete the proposed multi-task of intersection re-identification mainly due to our elaborately designed network structure. We compared our network with state-of-the-art place recognition algorithms and the results demonstrated its superior performance, more than 12% recognition precision gain. For generalization and robustness in traffic environment, we consider the rain-haze model for data augmentation. It shows that the data augmentation brings a part of performance gain in re-ID task, which demonstrates the positive effect by improving the diversity of training data. For the lack of intersection re-identification datasets, we propose a carefully-labeled large-scale intersection dataset, called “RobotCar Intersection”, which includes 36,588 images of eight intersections in different seasons and different day time. We also captured 3340 images of eight campus intersections to verify our re-ID approach on panorama modality and our expansion strategy of topology map. The related results show that our proposed method can detect and re-identify new intersection images.

In the future work, we plan to use attention mechanism, temporal characteristics, uncertainty estimation, fusion with GPS signals, etc., to improve the performance of our framework on intersection re-identification. Moreover, we plan to increase the intersection data by further recording real scene images as well as by generating virtual intersection scenes from simulation platforms and improved data augmentation models. To form a complete updating mechanism for intersection topology map, improved intersection detection is considered to fuse into our method.

## Figures and Tables

**Table 1:** Five different configurations for comparative experiments.

	1B	2B	FOC	TRI	AUG
1B + TRI + AUG	✓			✓	✓
2B + FOC		✓	✓		
2B + FOC + AUG		✓	✓		✓
2B + TRI		✓		✓	
2B + TRI + AUG		✓		✓	✓

**Table 2:** Precision of compared methods with baselines under different environmental conditions.

Methods	Total	Day	Night	Sunny	Overcast	Rainy
BOW [17]	0.689	0.736	0.311	0.685	0.746	0.544
ConvNet [22]	0.797	0.798	0.788	0.787	0.808	0.769
SiameseNet [24]	0.844	0.849	0.798	0.834	0.867	0.775
1B + TRI + AUG	0.964	0.963	0.972	0.956	0.968	0.893
2B + TRI	***0.971***	0.970	***0.989***	0.970	***0.973***	0.948
2B + TRI + AUG	***0.971***	***0.971***	***0.989***	***0.972***	0.972	***0.956***

**Table 3:** Precision of compared methods on the RobotCar Intersection data fused with images generated by rain and haze models.

Methods	1B + TRI + AUG	2B + FOC	2B + FOC + AUG	2B + TRI	2B + TRI + AUG
Precision	0.933	0.906	0.958	0.901	***0.963***

**Table 4:** Precision of compared methods in low traffic flow.

Methods	Total	Day	Night	Sunny	Overcast	Rainy
BOW [17]	0.728	0.743	0.349	0.652	0.777	0.356
ConvNet [22]	0.853	0.853	0.796	0.878	0.846	0.812
SiameseNet [24]	0.887	0.887	0.854	0.881	0.896	0.803
1B + TRI + AUG	0.975	0.975	0.968	0.983	0.976	0.931
2B + TRI	***0.982***	***0.982***	0.987	0.982	***0.984***	0.940
2B + TRI + AUG	***0.982***	***0.982***	***0.989***	***0.986***	0.983	***0.941***

**Table 5:** Precision of compared methods in high traffic flow.

Methods	Total	Day	Night	Sunny	Overcast	Rainy
BOW [17]	0.664	0.639	0.105	0.644	0.691	0.307
ConvNet [22]	0.738	0.738	0.745	0.707	0.763	0.713
SiameseNet [24]	0.861	0.861	0.910	0.887	0.887	0.690
1B + TRI + AUG	0.955	0.955	***0.980***	0.958	0.959	0.927
2B + TRI	***0.958***	0.957	***0.980***	***0.970***	***0.967***	0.911
2B + TRI + AUG	***0.958***	***0.958***	***0.980***	0.961	0.961	***0.928***

**Table 6:** Precision of proposed method under panoramic image modality in dealing with existed and new intersection images, which corresponds to the two main tasks in this chapter.

Task	Intersection ID	Attribute ID	Global ID
existed intersection re-ID (task 1)	0.985	0.987	0.998
new intersection detection (task 2)	-	0.855	0.990
new intersection re-ID (task 2)	-	0.855	0.991
all intersection re-ID (task 2)	-	0.924	0.996
